# Case Report: Taking action or standing by: managing a preterm neonate at the risk of neonatal varicella by metagenomic next-generation sequencing

**DOI:** 10.3389/fped.2024.1345338

**Published:** 2024-07-19

**Authors:** Haiting Li, Xiyang Chen, Jie Li, Linlin Chen, Xixi Liu, Caie Chen, Dengpan Xie, Yunqin Chen, Junhui Yuan, Enfu Tao

**Affiliations:** Department of Neonatology and NICU, Wenling Maternal and Child Health Care Hospital, Wenling, Zhejiang, China

**Keywords:** neonatal varicella, metagenomic next-generation sequencing, infection, diagnosis, prediction

## Abstract

Neonatal varicella is indeed a rare condition, and most infants born to mothers with varicella have a good prognosis. However, in exceptional cases, neonatal varicella can be life-threatening, particularly for preterm infants. Therefore, it is vital to make an early diagnosis or predict the risk of neonatal varicella to ensure prompt treatment and improve prognosis. This report made an effort to early predict neonatal vericalla by using metagenomic next-generation sequencing (mNGS) in a preterm infant who was at risk for vericalla infection. A preterm infant born from a mother with varicella with symptom onset at 8 days before delivery, putting the infant at risk for varicella infection. Importantly, the patient develop pneumonia and pneumothorax, and neonatal vericella was suspected. Fortunately, the use of mNGS for testing the varicella gene in the serum promptly ruled out varicella zoster virus (VZV) infection in the patient, as indicated by a negative mNGS result. Subsequent follow-up, which included a 14-day stay in the hospital followed by an additional 7 days at home, confirmed this finding. Throughout this period, the patient did not exhibit any rash or other symptoms associated with varicella. Therefore, the novel approach of using mNGS allows neonatologists to predict and promptly address potential neonatal infections. This early detection is crucial, as delayed diagnosis or treatment could pose life-threatening risks, as exemplified by the case of neonatal varicella. In such cases, neonatologists can take proactive measures instead of standing by for at-risk neonates. Furthermore, given the severity of neonatal varicella as a life-threatening condition, the early exclusion of subsequent varicella infection by mNGS can offer reassurance to both family members and healthcare professionals.

## 1 Introduction

Infectious varicella (chickenpox) is a highly contagious viral disease caused by varicella zoster virus (VZV) (1). Varicella and herpes zoster (HZ) are two different contagious diseases caused by the same virus, VZV (2). Primary VZV infection develops in childhood, causing chickenpox, and later in life, a latent infection develops into HZ (3). However, neonates are a special population in which infection by VZV may cause different outcomes, such as congenital varicella syndrome, varicella, HZ, and even neonatal death (4). These outcomes are closely related to the time when mothers were infected by VZV during pregnancy. The mortality rate of neonatal varicella was up to 20% if the mother developed symptoms of varicella between 4 and 5 days before and 2 days after delivery and neonates developed rash at 5–12 days of life (5).

Early prediction of neonatal varicella would be important for proper management, which can not only decrease the severity of the disease but also effectively reduce perinatal varicella incidence (6). Unfortunately, reported cases of neonatal varicella that led to death were not diagnosed early. Early diagnosis of neonatal varicella is elusive. Neonates infected by VZV were usually asymptomatic at birth due to its long incubation period, between 10 and 14 days and a maximum of 21 days, and discharged to home as normal newborns (6) but gradually presented vesicular symptoms and then deteriorated rapidly. The VZV can invade the lungs, liver, and central nervous system, finally leading to respiratory and circulatory failure and neonatal death (4, 6). Furthermore, prophylaxis of VZV—immunoglobulin and acyclovir cannot completely prevent neonatal varicella and varicella-induced death (6). Moreover, some physicians lack awareness and recognition of neonatal varicella (6). Due to these factors, the diagnosis and treatment of neonatal varicella are frequently delayed, leading to a bleak prognosis for high-risk neonates.

The metagenomic next-generation sequencing (mNGS), which focuses on the whole genome sequence of pathogens, is widely employed for clinical diagnosis (7, 8). Likewise, it has been extensively utilized for diagnosing neonatal infectious diseases (9–11). This report presents a case of a preterm infant who was born from a mother with varicella onset 8 days before delivery and was at risk for neonatal varicella. Neonatal varicella was predicted through mNGS. Fortunately, the mNGS was negative, and the patient was not infected by VZV. This case offers a reference plan for the early proactive management of vulnerable newborns and may contribute to enhancing the prognosis of at-risk neonates.

## 2 Case description

**Figure 1:**
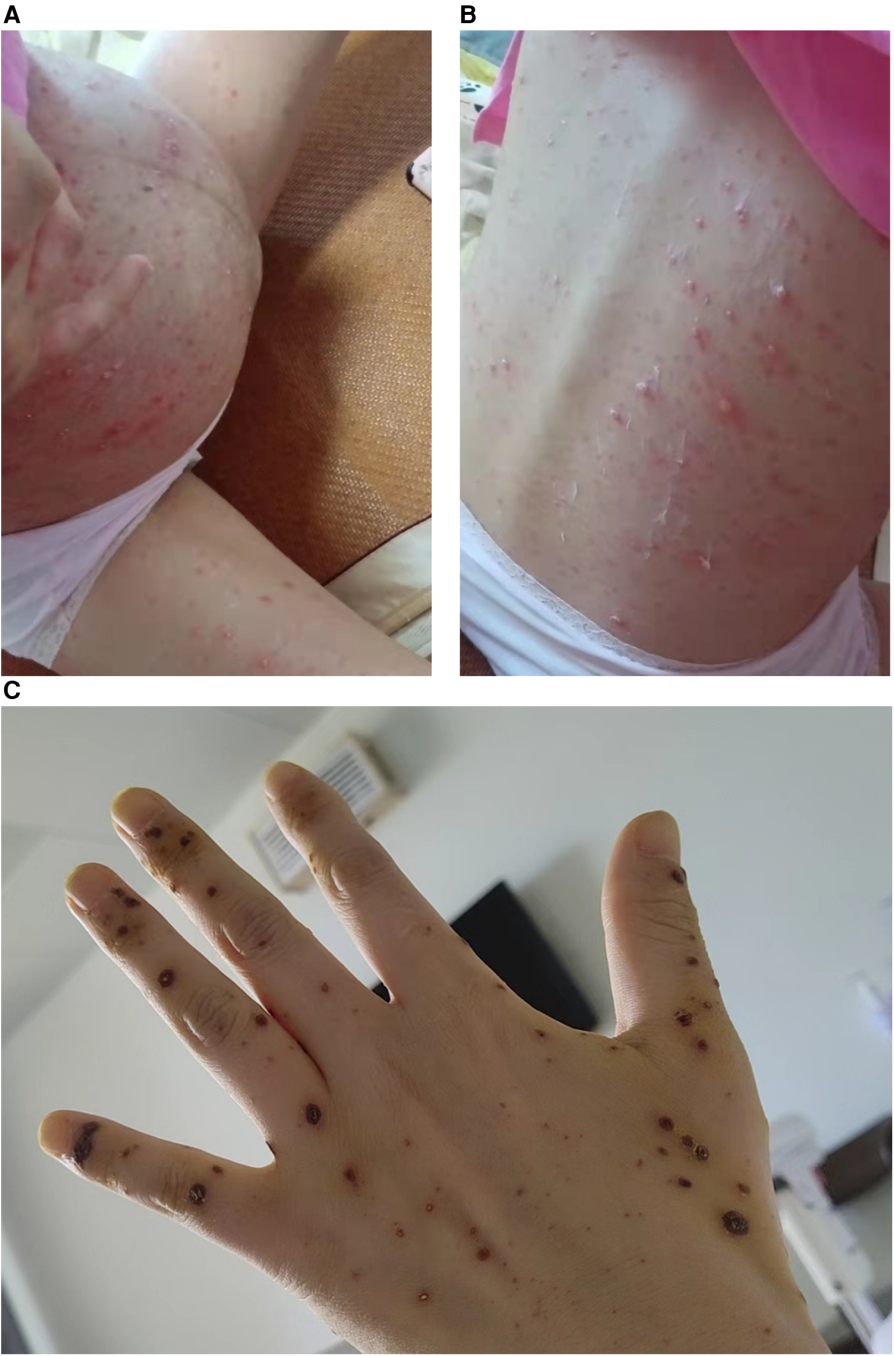
The skin manifestations of the mother with varicella infection. (**A**, **B**) The mother exhibits a widespread rash of vesicles. (**C**) The vesicles are healing and forming scabs.

A male preterm infant with a gestational age of 36 5/7 weeks and birth weight of 2,950 g was delivered from a mother who presented systemic vesicular rash 8 days before delivery and scab formation during delivery (Figure 1). Epidemiological investigation revealed the mother's close contact with the infant's father, who had varicella. The infant's father was in close contact with the infant's grandfather, who had HZ 3 weeks ago. The infant was vaginally delivered with no complications. He had no rash at birth.

**Figure 2:**
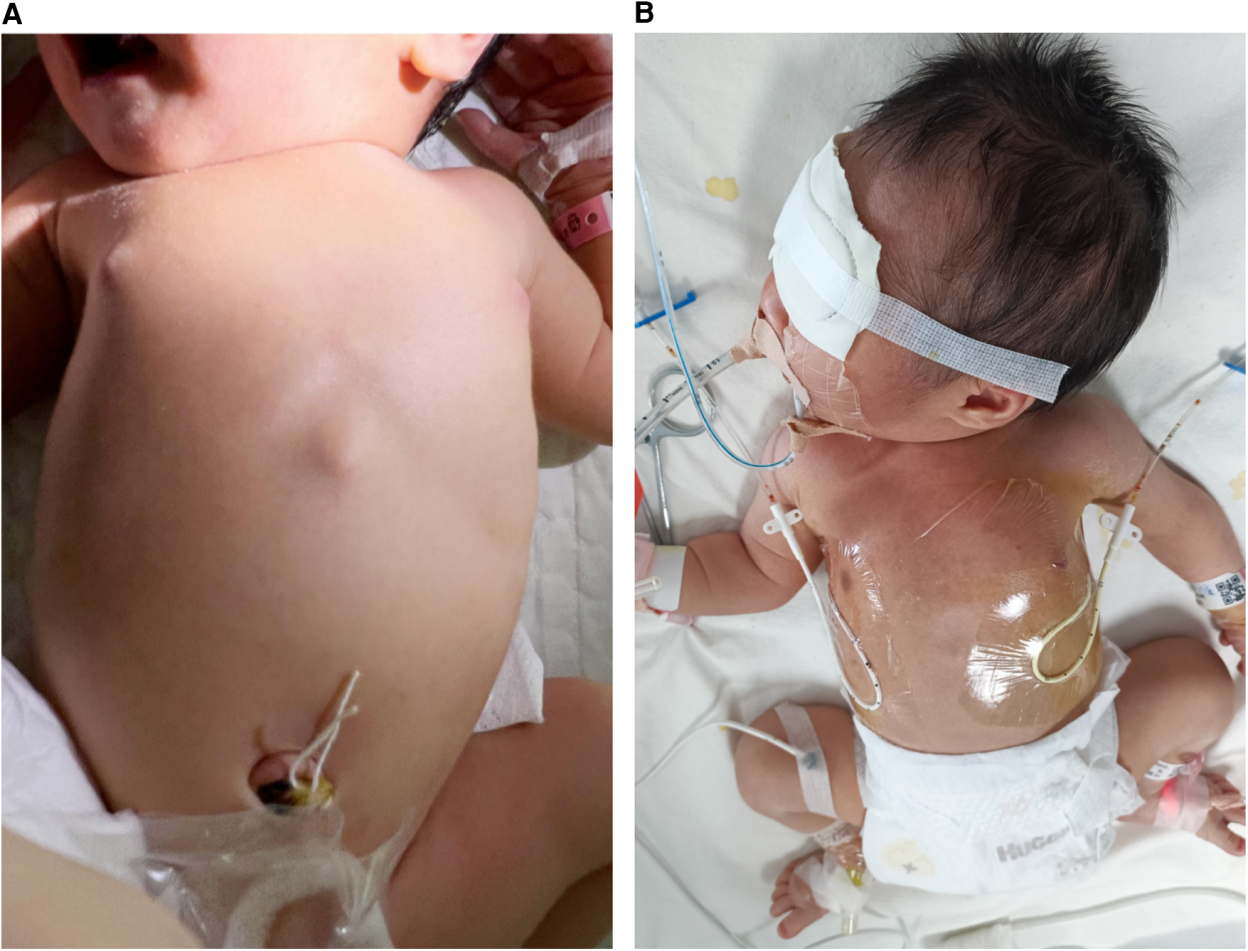
The preterm patient was at risk for neonatal varicella. (**A**) He had no rash at birth. (**B**) The patient was receiving closed-chest drainage, as well as invasive mechanical ventilatory support with high-frequency oscillatory ventilation.

The infant was admitted to the department of neonatology due to preterm birth and high risk for neonatal varicella. On examination, vital signs were stable, with normal cardiopulmonary auscultation, a flat and soft abdomen, absence of signs of hepatosplenomegaly, 2 s of capillary refill time, and absence of rash (Figure 2A).

**Figure 3:**
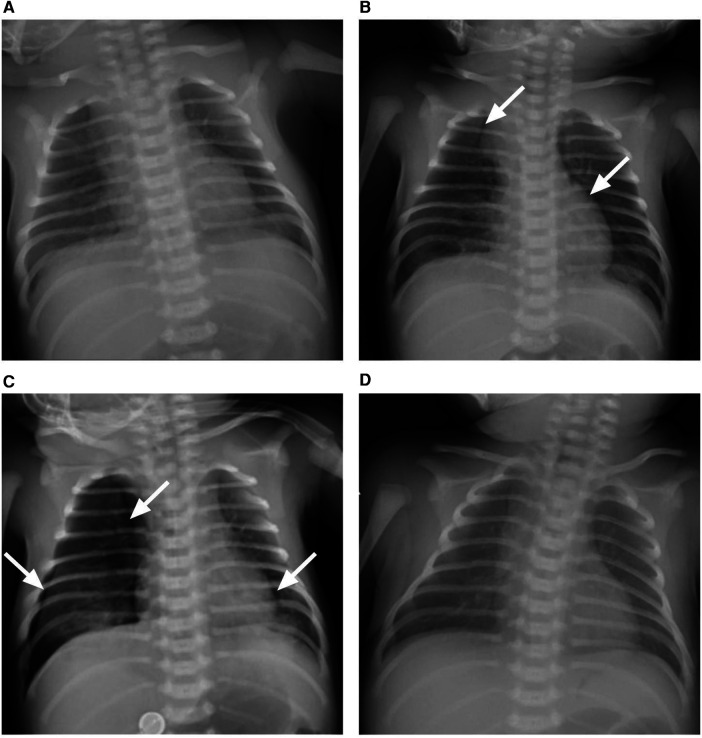
The chest x-ray presentation of the patient. (**A**) Bilateral pulmonary exudative lesions were found on admission, revealing pneumonia. (**B**) Lucency shadows were observed at two sides of para-mediastinum as marked with white arrows on day 2 after admission, suggesting pneumothorax. (**C**) Pneumothorax deteriorated, and the right lung was remarkably compressed as marked with white arrows on day 3 after admission; (**D**) bilateral pulmonary exudative lesions and pneumothorax were absorbed on day 10 after admission.

Intravenous accesses were established by nurses in anticipation of the possible administration of medication. Laboratory investigations of whole blood count, routine stool and urine tests, and blood biochemistry did not reveal remarkable abnormalities on admission. However, bedside chest radiography suggested neonatal pneumonia (Figure 3A). Empirical intravenous antibiotic therapy with penicillin was initiated. Intravenous acyclovir (20 mg/kg, q8 h) and immunoglobulin (400 mg/kg, qd) were used as prophylaxis for possible VZV infection.

On day 2 after admission, the patient presented shortness of breath, and the oxygen saturation was approximately 90%. A re-examination of the bedside chest radiograph revealed the progress of pneumonia and the presence of pneumothorax (Figure 3B). Infectious pneumonia was not completely excluded, and antibiotics of cefoperazone–sulbactam in combination with penicillin were used to cover both gram-negative and gram-positive bacteria. Blood gas analysis revealed a partial pressure of oxygen of 52.8 mmHg, suggesting hypoxemia. High-flow nasal oxygen (HFNO) was implemented for respiratory support, but tachypnea was not improved. A re-examination of the bedside chest radiograph indicated progressive pneumothorax (Figure 3C). Closed-chest drainage and high-frequency oscillatory ventilation immediately replaced HFNO as respiratory support (Figure 2B). Simultaneously, mNGS of the blood sample were detected for pathogen diagnosis, such as VZV, after informed written consent was obtained from the parents. The result of the mNGS was negative on day 3 after admission, implying no pathogenic microorganism infection in the blood. Moreover, the blood culture was also negative. The patient gradually improved, the closed thoracic drainage tube was removed, and mechanical ventilation retreated. A re-examination of the complete blood count revealed a normal hemogram, and procalcitonin and hypersensitive C-reactive protein levels were in the normal range. Antibiotics were discontinued after 5 days of use. Intravenous immunoglobulin was given over three consecutive days, and intravenous acyclovir was administered for the same duration until a negative result mNGS. Serology testing (enzyme linked immunosorbent assay, Virion-Serion Biotechnology, Germany) of the neonate revealed a positive anti-VZV IgG antibody and a negative anti-VZV IgM antibody. A re-examination of the bedside chest radiograph revealed that bilateral pulmonary exudative lesions and pneumothorax were absorbed on day 10 after admission (Figure 2D).

The final diagnosis for the patient was neonatal pneumonia, pneumothorax and hypoxemia. After receiving acyclovir and IVIG for VZV prophylaxis, followed by penicillin and cefoperazone–sulbactam antibiotics for infection control, along with high-frequency oscillatory ventilation and closed-chest drainage, the patient's condition has improved gradually. The patient stayed in the hospital for 14 days to monitor the risk of neonatal varicella. Finally, the infant was discharged and was well during the 21-day follow-up. The patient did not develop any rash from birth to 21 days of life. At the 3-month, 6-month, and 1-year follow-up visits, the patient's condition remained favorable, with no adverse neurological outcomes observed. The overview of the clinical course for the premature infant at risk of neonatal varicella was summarized in Table 1. The main laboratory test results for the premature infant at risk of neonatal varicella were summarized in Table 2.

**Table 1:** Summary of the clinical course for the premature infant at risk of neonatal varicella.

Age	Clinical findings	Treatment
Day 1	Stable vital signs; no rash observed; bedside chest radiography suggests neonatal pneumonia	Empirical intravenous antibiotic therapy with penicillin; Prophylactic intravenous administration of acyclovir (20 mg/kg, q8 h) and immunoglobulin (400 mg/kg, qd) for possible VZV infection
Day 2	Shortness of breath; oxygen saturation approximately 90%; pneumonia progression; pneumothorax	Antibiotic therapy: cefoperazone–sulbactam + penicillin; closed-chest drainage and high-frequency oscillatory ventilation
Day 3	Negative mNGS result	Discontinue acyclovir use
Day 10	Bilateral pulmonary exudative lesions and pneumothorax were absorbed	Monitoring vital signs and observing for the presence of rash
Day 14	Stable vital signs; no rash observed	Discharge
Day 21	Stable vital signs; no rash observed	Follow-up at home

VZV, varicella zoster virus; mNGS, metagenomic next-generation sequencing.

**Table 2:** Summary of laboratory investigations for the premature infant at risk of neonatal varicella.

Investigation	Results	Reference range
mNGS	Negative	Negative
Blood culture	Negative	Negative
Complete blood count		
White blood cell, ×10^9^/L	10.8	15–20
Neutrophil percentage, %	58.7	50–75
Lymphocytes percentage, %	31.7	20–40
Platelet, ×10^12^/L	344	100–300
Hypersensitive C-reactive protein, mg/L	<0.5	0–5.0
Procalcitonin, ng/ml	0.0722	<0.5
Serology testing		
Anti-VZV IgG antibody	Positive	Negative
Anti-VZV IgM antibody	Negative	Negative

mNGS, metagenomic next-generation sequencing; VZV, varicella zoster virus.

## 3 Discussion

Neonatal varicella is a rare yet potentially fatal condition, and the timely and accurate prediction of this condition remains challenging. In this report, we employed mNGS to predict neonatal varicella in a preterm infant at risk of varicella infection.

Typically, neonatal varicella is considered a benign but potentially fatal condition. A reported case highlighted the severity (6), where a newborn, initially asymptomatic, rapidly deteriorated after discharge, leading to respiratory distress, shock, and eventual demise on day 15. This case emphasizes the critical window when the mother exhibits varicella symptoms as crucial in determining neonatal outcomes. Neonates born to mothers who contract varicella within 5 days before delivery and up to 2 days after delivery face the highest risk of VZV infection and neonatal mortality. This heightened risk is attributed to the insufficient time available for the mother to generate and transmit protective anti-VZV IgG antibodies to the neonate through the placenta (4). Furthermore, this case underscores the critical importance of early diagnosis in neonatal varicella. Newborns may not manifest symptoms at birth, yet the disease can progress to severe consequences. Early identification of the illness in infants enables prompt implementation of treatment and monitoring, potentially averting neonatal mortality. Regrettably, there is currently a lack of published literature specifically addressing this matter, emphasizing the urgency to develop a novel approach for the early prediction of VZV infection.

Recently, mNGS of cerebrospinal fluid (CSF) has proven valuable in diagnosing central nervous system infections with VZV in adult patients (12, 13). Moreover, mNGS has successfully diagnosed VZV infection in a child (14). Experts have reached the consensus that there is ample literature data supporting the efficacy of mNGS in testing for varicella and other viruses in children (15). Zhu et al. demonstrated that using mNGS on CSF is more sensitive in detecting VZV compared to CSF VZV polymerase chain reaction (PCR) and antibody tests in adults. Furthermore, it offers the advantage of identifying unexpected pathogens (13). In another study, Wu et al. reported that 36.4% (4 out of 11) of patients tested positive for cytomegalovirus (CMV) through quantitative PCR, while mNGS achieved a 100% detection rate for CMV (16). In addition, Zhang et al. suggested that mNGS provided microbial findings for various neonatal infectious diseases, including pneumonia, sepsis, and meningitis (11). These research findings strongly advocate mNGS as an excellent diagnostic tool for neonatal infectious diseases, including neonatal varicella. Notably, there is a gap in the literature regarding mNGS for the detection of VZV infection in neonates. To the best of our knowledge, our report represents the inaugural use of mNGS for the early prediction of neonatal varicella infection. This pioneering approach introduces a new dimension to the early diagnosis of neonatal varicella, potentially contributing to improved outcomes for the at-risk population.

Of note, this report has several limitations. First, it is merely a case report. Secondly, the neonate in this case is born to a mother who exhibited varicella symptoms 8 days before delivery. This suggests that the neonate has sufficient time to acquire protective antibodies from the mother. Hence, further research is required to explore the potential role of mNGS in the early prediction of neonatal varicella among infants born to mothers experiencing varicella onset within 5 days before and 2 days after delivery. Nonetheless, this represents the inaugural attempt to apply mNGS to neonatal varicella, providing novel perspectives and methodologies for the future diagnosis of neonatal varicella.

## 4 Conclusions

A preterm infant born from mothers who develop varicella more than 5 days before delivery may not develop neonatal varicella. The utilization of mNGS demonstrates its value in the early diagnosis and prediction of neonatal varicella, particularly when the infant is at risk for varicella infection. This diagnostic method can help healthcare providers identify the condition promptly, allowing for timely intervention when needed. Additionally, the potential importance of mNGS extends beyond neonatology, offering significant benefits in other clinical scenarios. For example, it can be particularly crucial in the management of immunocompromised children, such as those with cancer or who have undergone organ transplants, who are accidentally exposed to active varicella or HZ.

## Data Availability

The original contributions presented in the study are included in the article/Supplementary Material, further inquiries can be directed to the corresponding author.
